# Increased mitochondrial ATP production capacity in brain of healthy mice and a mouse model of isolated complex I deficiency after isoflurane anesthesia

**DOI:** 10.1007/s10545-015-9885-x

**Published:** 2015-08-27

**Authors:** Ganesh R. Manjeri, Richard J. Rodenburg, Lionel Blanchet, Suzanne Roelofs, Leo G. Nijtmans, Jan A. Smeitink, Jacques J. Driessen, Werner J. H. Koopman, Peter H. Willems

**Affiliations:** Department of Biochemistry, Radboud Institute for Molecular Life Sciences, Radboud University Medical Centre, 286 Biochemistry, P.O. Box 9101, NL-6500 HB Nijmegen, The Netherlands; Department of Pediatrics, Nijmegen Centre for Mitochondrial Disorders, Radboud University Medical Centre, 804 Pediatrics, P.O. Box 9101, NL-6500 HB Nijmegen, The Netherlands; Department of Anesthesiology, Nijmegen Centre for Mitochondrial Disorders, Radboud University Medical Centre, 549 Anesthesiology, P.O Box 9101, NL-6500 HB Nijmegen, The Netherlands

## Abstract

We reported before that the minimal alveolar concentration (MAC) of isoflurane is decreased in complex I-deficient mice lacking the NDUFS4 subunit of the respiratory chain (RC) (1.55 and 0.81 % at postnatal (PN) 22–25 days and 1.68 and 0.65 % at PN 31–34 days for wildtype (WT) and CI-deficient KO, respectively). A more severe respiratory depression was caused by 1.0 MAC isoflurane in KO mice (respiratory rate values of 86 and 45 at PN 22–25 days and 69 and 29 at PN 31–34 days for anesthetized WT and KO, respectively). Here, we address the idea that isoflurane anesthesia causes a much larger decrease in brain mitochondrial ATP production in KO mice thus explaining their increased sensitivity to this anesthetic. Brains from WT and KO mice of the above study were removed immediately after MAC determination at PN 31–34 days and a mitochondria-enriched fraction was prepared. Aliquots were used for measurement of maximal ATP production in the presence of pyruvate, malate, ADP and creatine and, after freeze-thawing, the maximal activity of the individual RC complexes in the presence of complex-specific substrates. CI activity was dramatically decreased in KO, whereas ATP production was decreased by only 26 % (*p* < 0.05). The activities of CII, CIII, and CIV were the same for WT and KO. Isoflurane anesthesia decreased the activity of CI by 30 % (*p* < 0.001) in WT. In sharp contrast, it increased the activity of CII by 37 % (*p* < 0.001) and 50 % (*p* < 0.001) and that of CIII by 37 % (*p* < 0.001) and 40 % (*p* < 0.001) in WT and KO, respectively, whereas it tended to increase that of CIV in both WT and KO. Isoflurane anesthesia increased ATP production by 52 and 69 % in WT (*p* < 0.05) and KO (*p* < 0.01), respectively. Together these findings indicate that isoflurane anesthesia interferes positively rather than negatively with the ability of CI-deficient mice brain mitochondria to convert their main substrate pyruvate into ATP.

## Introduction

Inhaled anesthetics such as isoflurane and sevoflurane are extensively used in clinical practice but much concern remains regarding their possible detrimental effects, particularly on the developing brain (Loepke and Soriano 2008; Hays and Deshpande 2011; Chiao and Zuo 2014). Recent work shows that these anesthetics can induce mitochondrial fission in the developing brain, suggesting a mitochondrial component in the process of anesthesia-induced brain damage (Boscolo et al 2013).

Further evidence for a putative role of mitochondria in the process of anesthesia-induced brain damage comes from the observation that children with a deficiency of complex I (CI), but not complex III (CIII), of the respiratory chain (RC), are hypersensitive to volatile anesthetics (Morgan et al 2002; Driessen et al 2007). However, it is still debated whether these anesthetics put CI-deficient children at increased risk of neurological complications (Niezgoda and Morgan 2013). Recent studies with mice genetically engineered to lack the NDUFS4 subunit of CI of the RC (referred to herein as “CI-deficient KO mice”) corroborate the finding that the sensitivity to volatile anesthetics is increased in CI deficiency (Quintana et al 2012a, b; Roelofs et al 2014).

Regarding a putative role of CI in the mechanism of action of volatile anesthetics, studies investigating the effects of direct application of these anesthetics to intact and broken mitochondria conclude that low concentrations reversibly inhibit the oxidation of CI-, but not CII-, linked substrates (Miller and Hunter 1970; Harris et al 1971). Another line of evidence supporting a direct action of volatile anesthetics on CI comes from studies with *C. elegans*. This organism does not possess specialized respiratory systems and complex circulatory organs and relies entirely on the diffusion of gases across the gut lumen and the cuticle (Van Voorhies and Ward 2000). Loss-of-function mutations in CI, but not CII, genes, render these worms hypersensitive to volatile anesthetics in terms of immobility induction (Kayser et al 1999; Falk et al 2006). Analysis of several CI-deficient worm strains with different oxidation rates of CI-linked substrates revealed that the anesthetic sensitivity increased with decreasing oxidation rate (Falk et al 2006). In agreement with these observations, isoflurane was demonstrated to bind to a site distal to the flavoprotein subcomplex of CI (Kayser et al 2011). Taken together, these studies indicate that volatile anesthetics bind to CI to reduce its activity and that this inhibitory effect is increased by loss-of-function mutations in CI.

The RC generates the proton motive force used by CV (F0F1-ATP synthase) to produce ATP and with the activity of CI being reduced in CI-deficient KO mice and CI being a direct target of volatile anesthetics, it is speculated that these anesthetics reduce brain mitochondrial ATP production to a much larger extent in these KO mice than in WT mice. As a consequence, brain ATP levels would be much more decreased in anesthetized KO mice thus explaining their increased sensitivity to volatile anesthetics (Kayser et al 2004). To test this idea, we determined the maximal rate of ATP production and the maximal activity of the individual RC complexes in a whole brain mitochondria-enriched fraction from the WT and CI-deficient KO mice described in the previous study (Roelofs et al 2014).

We show that in vivo treatment of WT mice with isoflurane decreased the maximal activity of CI, while it increased maximal ATP production. Isoflurane anesthesia also increased maximal ATP production in CI-deficient KO mice. In both WT and KO mice, the effect of isoflurane on brain mitochondrial ATP production was accompanied by an increase in CII and CIII maximal activity and a tendency to increase for CIV. Our data show that isoflurane anesthesia improves rather than worsens the ATP generating ability of brain mitochondria in both WT and CI-deficient KO mice.

## Materials and methods

All experiments were approved by the Regional Animal Ethics Committee (Nijmegen, The Netherlands) and performed under the guidelines of the Dutch Council for Animal Care. All efforts were made to reduce animal suffering and number of animals used in this study.

### Animals

This study uses the brains from the WT (*ndufs4*^+/+^) and KO (*ndufs4*^-/-^) mice included in our previous study on the anesthetic and respiratory depressant effects of isoflurane (Roelofs et al 2014). The genotype of the mice was confirmed by polymerase chain reaction and both male and female mice were included. Mice were group-housed at the central animal facility (CDL) of the Radboud University at 22 °C on a day and night rhythm of 12 h. The animals had *ad libitum* access to food and water and were fed on a standard animal diet (Ssniff GmbH, Soest, 76. Germany. V1534-300 R/M-H)*.*

### Isoflurane administration

WT (*n* = 5) and KO (*n* = 7) mice were subjected twice, i.e., at PN 22–25 and PN 31–34 days, to a well-established anesthesia protocol to determine the minimal alveolar concentration (MAC) of isoflurane (Roelofs et al 2014). Briefly, the isoflurane concentration was increased with steps of 0.2 % until the response to electrical stimulation of the hind paw was lost. When this point was reached the isoflurane concentration was decreased until return of the response. After the first MAC determination at PN 22–25 days, the animals were returned to their housing and determination of the MAC was repeated at PN 31–34 days.

### Tissue harvesting for biochemical analyses

Animals were sacrificed at PN 31–34 days by cervical dislocation. Isoflurane-treated mice were sacrificed immediately after determination of the MAC at PN 31–34 days. Whole brains were transferred to ice-cold SEF buffer (0.25 mol/L sucrose, 2 mmol/L EDTA, 10 mmol/L potassium phosphate, pH 7.4), minced with a Sorvall TC2 tissue chopper and homogenized with a glass/Teflon Potter Elvehjem homogenizer within 1 h of harvest. The homogenate was centrifuged at 600 g and a portion of the supernatant was used for measurement of the maximal rates of pyruvate oxidation and ATP production. The remainder of the 600 g supernatant was frozen in 10 μl aliquots in liquid nitrogen and kept at −80 ° C for maximal enzymatic activity measurements. The protein concentration was measured according to Rodenburg (Rodenburg 2011).

### Pyruvate oxidation and ATP production measurements

To determine the maximal rate of pyruvate oxidation, the freshly prepared 600 g supernatant was incubated with radiolabeled substrate ([1-14C] pyruvate). After 20 min the reaction was stopped and the amount of liberated radioactive CO_2_ (^14^CO_2_) was quantified (Janssen et al 2006). The assay medium (pH 7.4) contained K^+^- phosphate buffer (30 mM; source of Pi), KCl (75 mM), Tris (8 mM), K-EDTA (1.6 mM), P1,P5-Di (adenosine-5′) pentaphosphate (Ap5A; 0.2 mM), MgCl_2_ (0.5 mM), ADP (2 mM), creatine (20 mM), malate (1 mM), and [1-14C] pyruvate (1 mM). AP5A is a potent adenylate kinase inhibitor required to prevent interference of the adenylate kinase reaction with the levels of produced ATP, as well as with the excess of ADP required for this assay. For measurement of the maximal rate of ATP production, the same assay medium was used but with pyruvate instead of [1-14C] pyruvate. After 20 min, the reaction was stopped by addition of 0.1 M HClO_4_. The reaction mixture was centrifuged at 14,000 g for 2 min at 2 °C. To the supernatant, 1.2 vol (V/V) of 0.333 M KHCO_3_ was added, and this mixture was diluted twofold. The amount of ATP and phosphocreatine formed during the reaction were measured in the supernatant using a Konelab 20XT auto-analyzer (Thermo Scientific). Mitochondrial ATP production rate was corrected using a parallel assay in which residual glycolysis was blocked by arsenite (2 mM) (Janssen et al 2006).

### Respiratory chain enzyme assays

The liquid nitrogen frozen portion of the 600 g supernatant was thawed and used for measurement of the maximal activity of the complexes I (CI), II (CII), III (CIII), and IV (CIV) and citrate synthase (CS), as described by Rodenburg (Rodenburg 2011).

## Statistical analysis

Statistical analysis was performed using Prism 5 (GraphPad Software Inc., La Jolla, Ca). Normal distribution of the datasets was confirmed using Lilliefors test. Results were expressed as mean ± SD and comparisons between groups were performed using a two way analysis of variance (ANOVA) and Bonferroni’s post test. Statistical significance was set at (*p* < 0.05).

## Results

**Fig. 1:**
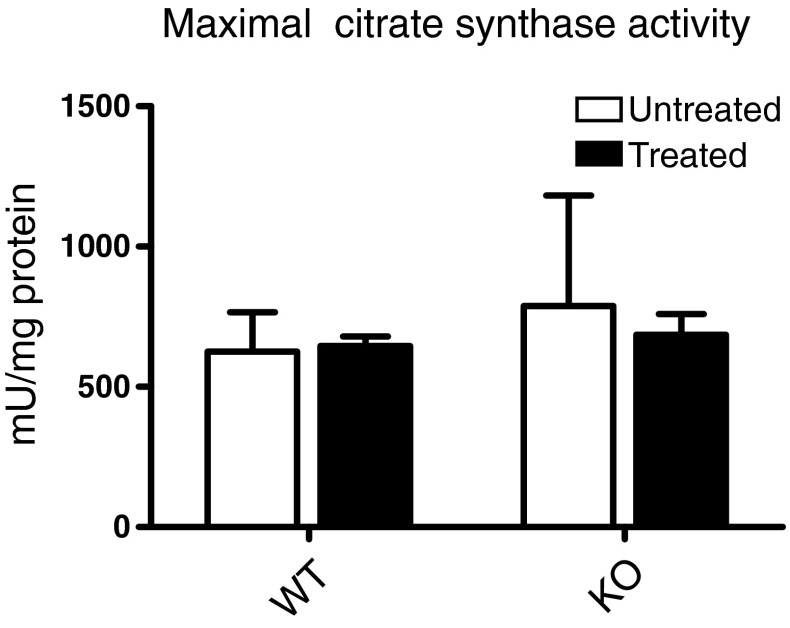
Effect of isoflurane on citrate synthase activity. Untreated and isoflurane-treated WT and KO mice were sacrificed at PD 31–34 and a mitochondria-enriched fraction was prepared from total brain. Citrate synthase activity was measured and expressed per mg protein. The data presented show the absence of any difference between the four experimental conditions. The data presented are the mean ± SD of 5 WT and 5 KO untreated and 5 WT and 7 KO isoflurane-treated

In mitochondrial enzyme diagnostics, the activity of CS, which is an indicator of mitochondrial mass, is used for normalization between mitochondria-enriched preparations (Rodenburg 2011). The CS activity per mg protein was the same for WT and KO mice and did not change upon isoflurane anesthesia (Fig. 1). In the remainder of this paper all values are expressed per mg protein.

**Fig. 2:**
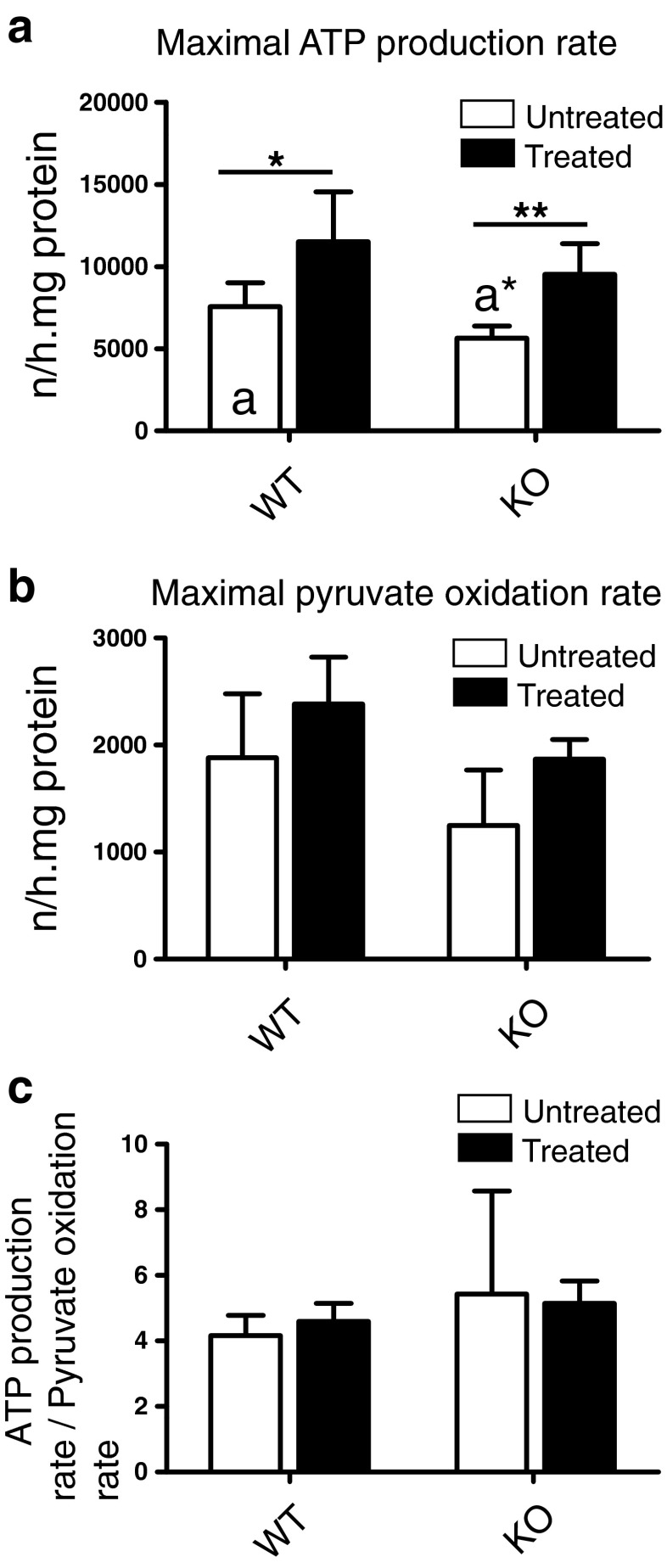
Effect of isoflurane on ATP production. Untreated and isoflurane-treated WT and KO mice were sacrificed at PD 31–34 and a mitochondria-enriched fraction was prepared from total brain. The rates of ATP production and pyruvate oxidation were measured at non-rate-limiting concentrations of pyruvate, malate, ADP, and creatine and expressed per mg protein. **a** The ATP production rate measured under these conditions was significantly decreased in untreated KO as compared to untreated WT (indicated with a* as compared with a). Isoflurane anesthesia significantly increased this rate in both WT and KO. **b** The maximal pyruvate oxidation rate tended to be decreased in untreated KO and increased in isoflurane-treated WT and KO. However, none of these differences reached statistical significance. **c** The ratio of the rate of ATP production to that of pyruvate oxidation, reflecting the coupling efficiency, was not significantly different between the different experimental conditions. The data presented are the mean ± SD of the number of animals indicated in the caption to Fig. 1. Statistical significance is displayed as * (*p* < 0.5) and ** (*p* < 0.01)

The maximal rate of ATP production was significantly decreased by 26 % in untreated KO as compared to untreated WT (Fig. 2a). Unexpectedly, isoflurane anesthesia significantly increased this rate in both WT and KO by 52 and 69 %, respectively. The maximal rate of pyruvate oxidation revealed a tendency to be lower in untreated KO as compared to untreated WT and isoflurane anesthesia tended to increase this rate in both WT and KO by 26 and 50 %, respectively (Fig. 2b). To evaluate the efficiency by which the oxidation of pyruvate was coupled to the production of ATP, we calculated the ratio of ATP production to pyruvate oxidation. The ratios obtained showed similar values for untreated and treated WT and KO mice (Fig. 2c).

**Fig. 3:**
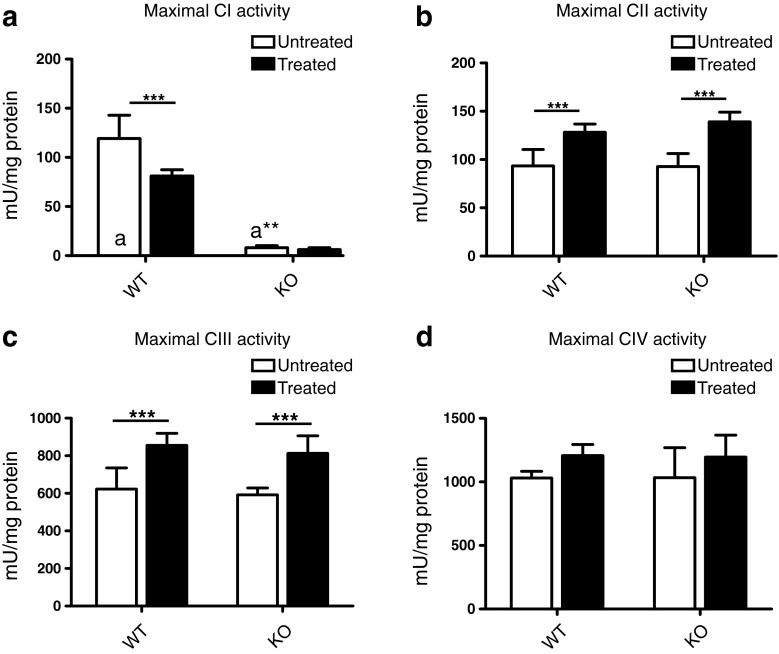
Effect of isoflurane on the enzymatic activities of the mitochondrial respiratory chain complexes. Untreated and isoflurane-treated WT and KO mice were sacrificed at PD 31–34 and a mitochondria-enriched fraction was prepared from total brain. The activities of the four respiratory chain complexes were measured under non-rate-limiting substrate conditions and expressed per mg protein. The values obtained reflect the maximum catalytic capacities of the complexes. **a** As expected, KO brain was virtually devoid of CI activity (indicated with a** as compared with a). Isoflurane anesthesia significantly decreased this activity in WT. **b** The activity of CII did not differ between WT and KO. Isoflurane anesthesia significantly increased this activity to the same extent in both WT and KO. **c** The activity of CIII was the same for WT and KO and also in this case isoflurane anesthesia increased this activity to the same extent in both WT and KO. **d** Also the activity of CIV was the same for WT and KO. Isoflurane anesthesia tended to increase this activity in both cases but this effect did not reach statistical significance. The data presented are the mean ± SD of the number of animals indicated in the caption to Fig. 1. Statistical significance is displayed as ** (*p* < 0.01) and *** (*p* < 0.001)

Analysis of the maximal activity of CI, revealed a significant decrease by 30 % in isoflurane-treated WT as compared to untreated WT (Fig. 3a). As expected, this activity was virtually absent in untreated KO and isoflurane treatment did not lead to any alteration. The maximal activity of CII, was similar between untreated WT and untreated KO (Fig. 3b). Isoflurane anesthesia significantly increased this activity by 37 and 50 % in WT and KO, respectively. The same results were obtained for CIII (Fig. 3c). Isoflurane anesthesia significantly increased the maximal activity of this complex by 37 and 40 % in WT and KO, respectively. For CIV, no difference in maximal activity was observed between untreated WT and untreated KO (Fig. 3d). Although isoflurane anesthesia tended to increase this activity in both WT (17 %) and KO (16 %), no statistical significance was reached.

## Discussion

Previous work with the NDUFS4 KO mouse model of human CI deficiency showed a decrease in respiratory rate (Quintana et al 2012a, b; Roelofs et al 2014) and heart rate (Quintana et al 2012a, b) in the later stages of the disease. The respiratory rate of the KO mice used in this study decreased from 130 at PN 22–25 days to 83 at PN 31–34 days (Roelofs et al 2014). This decrease in respiratory rate was paralleled by neurological complications that progressed with age (Quintana et al 2010). Although a decrease in respiratory rate suggests ATP shortage, other disease mechanisms remain to be considered, including increased production of reactive oxygen species (Koopman et al 2013) and triggering of innate immune responses (Yu et al 2015).

Here, we show that maximal ATP production from pyruvate and malate is 25 % decreased in a mitochondria-enriched fraction from KO mice brain at PN 31–34 days. The same observation was reached in another NDUFS4 KO mouse model (Leong et al 2012). At first glance, this relatively moderate decrease seems difficult to reconcile with the virtually complete absence of active CI in a freeze-thawed aliquot of this mitochondria-enriched fraction (see also, Kruse et al 2008 and Calvaruso et al 2012). However, there is evidence that CIII stabilizes NDUFS4-lacking CI to provide partial activity (Calvaruso et al 2012). This stabilization may be lost upon freeze-thawing of the mitochondria-enriched fraction. Intriguingly, isoflurane anesthesia of KO mice restored brain mitochondrial ATP production to WT levels. This result indicates that the underlying process does not involve irreversible damage, as is thought to occur at increased levels of reactive oxygen species. It is tempting to speculate that isoflurane improves the stabilization of NDUFS4-lacking CI by CIII.

The most intriguing observation of the present study is that isoflurane anesthesia increased rather than decreased brain mitochondrial ATP production in both WT and CI-deficient KO mice. To our best knowledge, this is the first report that describes such an effect of a volatile anesthetic, see also (Miro et al 1999). A recent in vivo study showed that isoflurane anesthesia decreased ATP levels in mouse brain (Wang et al 2015). Together, these data lead us to postulate that isoflurane acts outside the mitochondrion to reduce the supply of pyruvate, which is the main mitochondrial substrate in brain.

The increase in brain mitochondrial ATP production observed in anesthetized WT and KO mice was paralleled by a tendency to increase for the pyruvate oxidation rate, whereas the CS activity remained unaltered. This may suggest that pyruvate dehydrogenase is not the rate-limiting enzyme in the untreated condition. Isoflurane anesthesia significantly increased the activities of CII and CIII and tended to increase the activity of CIV. This may suggest that the activity of the RC is rate-limiting in the untreated condition and that isoflurane can simultaneously increase their activity by a hitherto unknown mechanism, which may, however, be triggered by ATP shortage (see above). In sharp contrast, the activity of CI was significantly decreased in anesthetized WT mice, indicating that this enzyme is not rate-limiting in the untreated condition.

Analysis of the ratio of ATP production to pyruvate oxidation revealed similar values for WT and CI-deficient KO mice regardless whether they were anesthetized or not. This result indicates, firstly, that the absence of the NDUFS4 subunit does not alter the coupling of pyruvate oxidation to ATP production and, secondly, that in vivo exposure to isoflurane does not alter this efficiency. Pyruvate oxidation is measured in the presence of an excess of pyruvate and malate, which is converted into oxaloacetate to trap acetyl-CoA, and an excess of ADP, which is converted into ATP. Under these conditions, a decreased efficiency of the coupling of pyruvate oxidation to ATP production would be indicative of an increased proton leak across the inner mitochondrial membrane. The present finding that in vivo exposure to isoflurane does not alter the coupling efficiency is of relevance since in vitro studies showed that direct addition of halothane to isolated mitochondria caused a limited uncoupling at concentrations between 0.5 and 2 % used clinically to achieve anesthesia (Miller and Hunter 1970).

Thus far, only inhibitory effects of volatile anesthetics on mitochondrial ATP production have been reported (Miller and Hunter 1970; Harris et al 1971). Available evidence indicates that volatile anesthetics act directly on CI (Kayser et al 2011) to decrease its activity (Miller and Hunter 1970; Harris et al 1971). Crucially, these studies employ direct application of the anesthetic to isolated mitochondria. For example, halothane, was shown to dose-dependently inhibit the rate of ADP-stimulated oxygen consumption in the presence of CI-, but not CII-linked substrates (Miller and Hunter 1970). Direct application of halothane to deoxycholate-treated mitochondria confirmed that CI, and not CII, was the primary target of the anesthetic (Harris et al 1971). The present study shows that the in vitro activity of CI was also decreased after in vivo exposure. The inhibitory effect in direct application studies was reversed in less than 5 min (Miller and Hunter 1970; Harris et al 1971), indicating that the mechanism of inhibition must be different from that in the present study. Our finding of a sustained effect of volatile anesthetics is corroborated by a recent study showing that 4 h of anesthesia at the larval stage of *C. elegans* caused a marked reduction of the chemotactic response at day 4 of life (Gentry et al 2013). Also in this study, it was observed that the degree of reduction was significantly more in worms with a loss-of-function mutation in a CI gene than in wild type worms.

Unfortunately, our anesthesia protocol does not allow to draw conclusions on whether the effects of isoflurane anesthesia at PN 31–34 days are acute or whether, and, if so, to which extent, they are a consequence of processes triggered during the first period of isoflurane anesthesia at PN 22–25 days. Long-term effects of volatile anesthetics have been reported in the literature and can be neuroprotective, as observed in a variety of animal stroke models, reviewed in (Burchell et al 2013) or neurotoxic, as evidenced by animal studies showing that volatile anesthesia can trigger widespread cell death in the neonatal developing brain (Jevtovic-Todorovic et al 2003; Yon et al 2005; Istaphanous et al 2011; Istaphanous et al 2013; Xiong et al 2013).

A limitation of our study is that we used a mitochondria-enriched fraction from whole brain homogenate. Since regional susceptibilities to mitochondrial dysfunction have been reported within the CNS (Leong et al 2012; Pinto et al 2012; Quintana et al 2012a, b), a further detailed investigation should involve specific regions of the brain. Thus, from the results presented in this study, we conclude that isoflurane exposure might preserve the ATP production capacity in brain mitochondria of CI-deficient KO mice and that the isoflurane hypersensitivity in these mice, is not a consequence of ATP deficits in the brain.
